# Bibliometric Analysis of Publication Activity in the Field of GIANT Cell Arteritis: A SCOPUS-based Study

**DOI:** 10.1055/a-1760-0340

**Published:** 2022-03-10

**Authors:** Syeda Beenish Bareeqa, Syeda Sana Samar, Sufiyan Kamal, Fizza Zaman Khan, Hafsa Shakeel, Kanza Ali, Syed Hasham Humayun, Syed Ijlal Ahmed

**Affiliations:** 1 Medical Graduate, Jinnah Medical and Dental College, Karachi, Pakistan; 2 Medical, Jinnah Sindh Medical University, Karachi, Pakistan; 3 Neurology Resident, Department of Neurology, Saint Louis University, Saint Louis, Missouri, United States

**Keywords:** bibliometric, citation classics, vasculitis, giant cell arteritis, temporal arteritis, polymyalgia rheumatic

## Abstract

**Objective** Giant cell arthritis (GCA) is a type of vasculitis which is more common in female gender and is closely associated with Polymyalgia rheumatic. One of its important complication include visual impairment. The burden of disease is expected to be very high by 2050 and there is a need to compile the data on most influential studies on GCA to define future strategy to deal with this dangerous disease. Bibliometrics is a statistical analysis of published literature that reflects the value and impact of a particular publication within the specific field. Aim of our study is identify the most significant contributors and their quality of contribution in the field.

**Method** We conducted this analysis utilizing SCOPUS database using different related MeSH terms. After a detailed screening, the list of top-50 articles were presented in the results in descending order of their ranks on the basis of their total number of citation. Most of our data comprises of publications from 1971–2012.

**Result** The top-50 most cited articles on GCA were published between 1971 and 2012 with the median number of citations 274 ranging from 598–187. *Annals of Internal Medicine* was the top ranked journal with 13 publications from the list. The highly ranked author based on the number of publications was *Hunder GG* (20 publications) with h-index of 40, retaining affiliation with Mayo Clinic, Rochester, United States. *Mayo Clinic* was the most frequently mentioned institute among the affiliations. The United States was found to be the most productive country rendering most of the articles (64%).

**Conclusion** Our bibliometric analysis on Giant cell arteritis identifies the information which may direct future research contributions, identify field experts, guide researchers to fill knowledge gaps, and assist in research fund allocation.

## Introduction

Giant-cell arteritis (GCA), also known as temporal arteritis, can be defined as an inflammatory vasculopathy of large to medium-size arteries. It is more common in Caucasian elderly females and is closely associated with Polymyalgia rheumatica. The incidence of GCA significantly increases after age of 50 years. This disease is most prevalent in Northern Europe and Minnesota-USA (≥20/100,000 people aging more than 50 years) with Japan being a territory with lowest rates of GCA (1.47/100,000 people aging more than 50 years). 1 2 According to a disease burden study conducted by Smit ED et al and company, it is predicted that there will be an expected increment of around 3 million more cases of GCA by the year 2050. Out of these 3 million new cases, at least half a million will be presenting with the complication of visual impairment. 3 Due to a high anticipated disease burden in next three decades, there is a need to compile and assess the most influential studies that has been done on GCA and redefine future strategies to prevent it.

Bibliometrics is a quantitative statistical analysis of published academic and scientific literature that reflects the value and impact of a particular article within the specific field. 4 Recently, it has been widely used as a tool to assess the characteristics of most influential data on particular topic using ‘Citation Classics’. Citation classics or the number of times an article is cited in the scholarly journals reflects the impact of author's scientific contribution and influence of an article on a distinct biomedical specialty. 5 There is a crucial ongoing debate on the significance of citation classics as a measure to assess the impact and influence of an article. Back in time, critical assessment by some scholars suggested few errors and bias that might have devalued the citation count as a method for analysis of scholarly productivity, however future researches found these errors as trivial and insignificant. Citation classics provides a scientific, transparent and sensitive criterion to compute the list of most impactful studies of each research area. 6

Citation analysis has been conducted on numerous different fields including respiratory system, 7 neurosurgery 8 and vascular surgery 9 however, after a detailed search of medical literature, no bibliometric analysis on Giant cell arteritis was found.

To the best of our knowledge, we've conducted the first ever citation analysis on GCA. The aim of our study is to identify the strengths, knowledge gaps, most contributing era and prominent scholars in the published research on GCA.

## Methodology

We conducted a citation analysis of studies published in the field of Giant Cell Arteritis. This analysis was conducted in November 2021 by using the Scopus Library Database ( www.scopus.com ) and we generated a list of top 50-most cited published articles on Giant Cell Arteritis. All the relevant articles having terms “Giant Cell Arteritis” OR “Temporal Arteritis” OR “Giant Cell Vasculitis” OR “Polymyalgia Rheumatica” within their titles, abstract and keywords were included in our search. After a substantial search, the initial list of relevant articles was categorized under “Cited by highest.” Concerning the risk of bias, two independent authors (SIA & SSS) generated two independent lists of top 50 most cited articles and a third independent author (SBB) analyzed both the lists to identify and eliminate any existing discrepancies. Agreement between reviewers was high (κ  =  0.96). Our literature search covered all the original and review articles, case reports and RCTs. However, we excluded those top cited studies which can be categorized under classifications, guidelines, editorials, conference papers and grants. There were no time period or abstracts availability limitation was applied in our search.

After a thorough screening, the list of top 50 articles was tabulated in descending order of their ranks on the basis of their total number of citations. Moreover, the average number of citations per year was calculated for each study by dividing the total number of citations by the number of years since its publication date. This highlighted the citation density per article on the basis of which we assigned a new ‘corrected rank’ to each study. Details of the citation classics are presented as supplementary data in Table S1 (online only). By using Scopus, all the pertinent data including top and impactful authors with their affiliation and number of their studies in the top-50 list and the top-journals with their impact factor of 2018 and country of origin was extracted and tabulated separately under each category respectively.

**Fig. 1:**
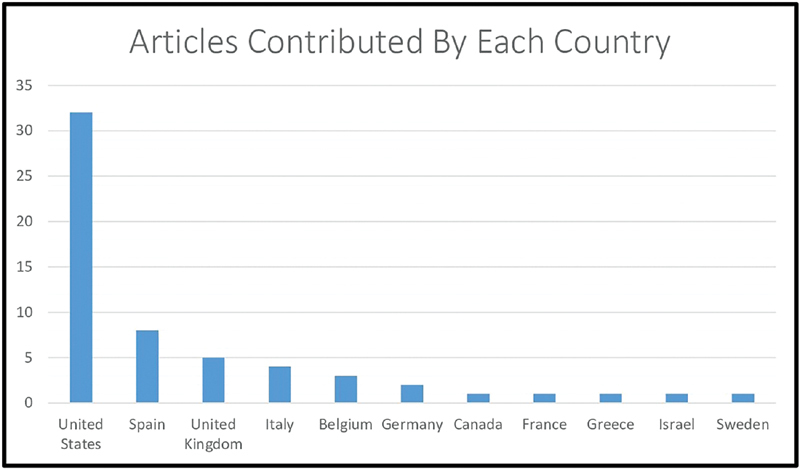
Distribution of articles by country of origin.

After examining each study, we subcategorized and charted them as under ‘diagnostic studies’, ‘management studies’, ‘epidemiological studies’, 'studies on clinical complications' and ‘prognosis studies’ and those studies which came under more than one of the above mentioned categories were categorized as ‘others’. From the oldest to the newest study in our list, we divided the timeline of 1971–2012 into a 10 year interval and charted them against the ‘number of articles published’ to identify the number of studies published per decade. Top-ranked journals were listed on the basis of having 3 or more publications in the list of most impactful 50 articles on GCA along with their impact factors of 2018. Leading authors with 4 or more publications within the top-50 most cited articles list along with their h-index, position within the article and affiliations were also presented in tabulated form. Details of top-ranked journals and top-ranked authors is given in Table 1 and Table 2 respectively. Article's country of origin was also extracted and illustrated in Fig. 1 .

**Table 1:** Top ranked journals with 3 or more publication on giant cell arteritis in top-50 list

Journal's Name	Number of Articles
**Annals of Internal Medicine**	13
**Arthritis And Rheumatism (continued as Arthritis and rheumatology)**	12
**Arthritis Care And Research**	4
**American Journal Of Ophthalmology**	3
**Annals Of The Rheumatic diseases**	3

**Table 2:** Top ranked authors with 4 or more publication within the list of top-50 most influential articles on GCA

Author Name	Total Number of Articles in list	Author Position in the article			Affiliation(s)
		Principle Author	Mentor Author	Other	
Hunder GG	20	1	7	12	Mayo Clinic, Rochester, United States
Weyand CM	7	3	2	2	VA Medical Center, United States
Goronzy JJ	6	0	4	2	VA Medical Center, United States
O'Fallon WM	4	0	1	3	Mayo Clinic, Rochester, United States
Salvarani C	4	3	0	1	University Degli studi Di Modena, Modena, Italy

**Fig. 2:**
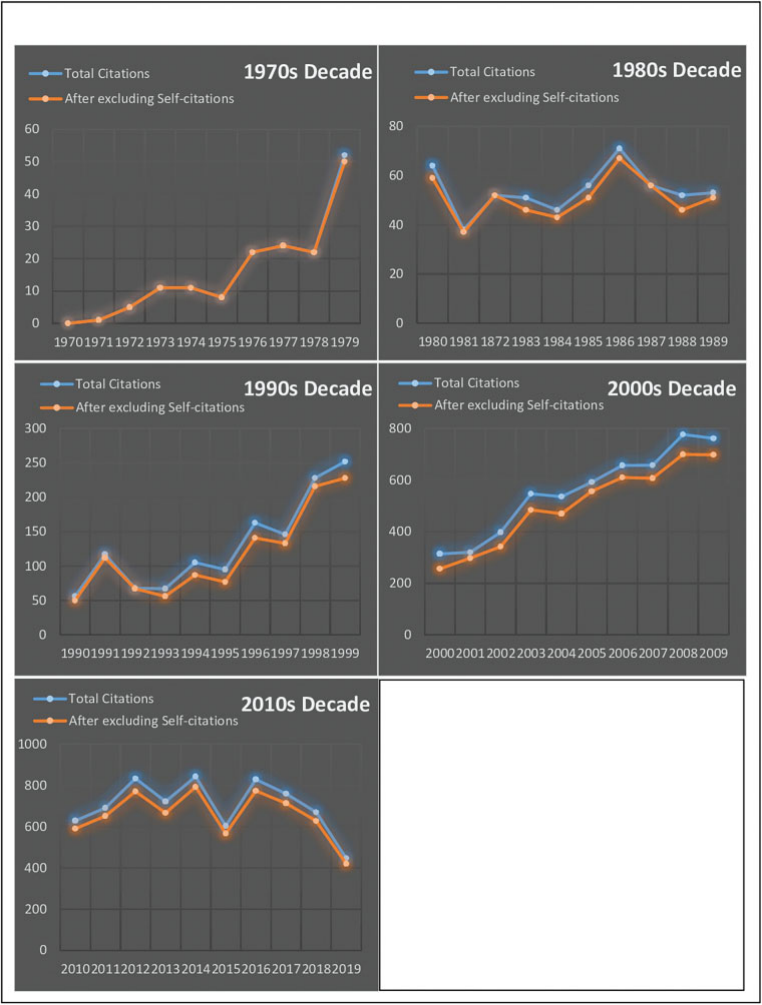
Graphical presentation of rise or decline in citation frequency through decades and difference between total number of citation and number of citations after excluding self-citations.

Scopus tool of ‘View Citation Overview’ was used to chart out the increase or decrease in the citation frequencies of GCA articles in top-50 list over the past 50 years. First, the total number of citation was counted and charted out on Fig. 2 . Then option of ‘Excluding Self-citation’ was applied and charted the citation count after self-citation exclusion. Details of citation frequencies is given in Fig. 2 .

IBM Statistical Package for Social Sciences (SPSS) version 21.0 was utilized to analyze the extracted data. Pearson product-moment correlation coefficient test was applied to analyze the linear relation between journal's impact factor and number of articles from top-50 list. Paired *t* -test was used to analyze the mean difference between citation frequencies of total citation obtained versus number of citations after excluding self-citation over the last five decades. A p-value of <0.05 was considered as significant.

## Results

There is a distinct variability in choosing the number of articles that will sufficiently represent the collection of most influential article on specific topic. For example, a bibliometric analysis conducted on spine related research choose to analyze only top-20 most cited articles meanwhile, another citation classic study conducted an analysis on top-100 most cited articles on the same topic. 10 11 Therefore, we decided after collaborative discussion that a citation analysis on top-50 most influential articles would be appropriate for GCA.

### Citation Metrics

**Fig. 3:**
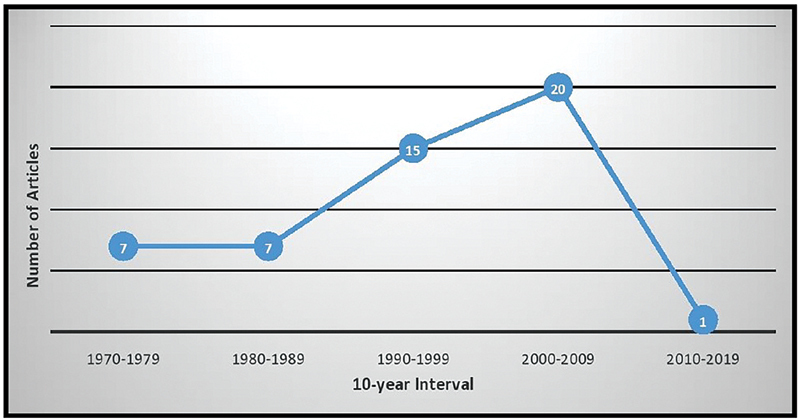
Distribution of articles on the basis of 10-years interval.

The top-50 most cited articles on GCA were published between 1971 and 2012 with the median number of citations 274 ranging from 598–187. The average citation density ranged from 4.81 to 44.5 citations per year. Corrected ranks on the basis of citation density were ranging from -31 to +45. Detail of article, original rank, citation classics, the average number of citations per year, and corrected rank is given in Supplementary Table S1 (online only). Most numbers of articles ( *n* = 20 articles) were published during the 10-year period of 2000–2009 with the highest mean number of citation ( *n* = 304.25). It is followed by a 10-year interval from 1990–1999 ( *n* = 15 articles) with 288.8 as mean number of citation. Details of the number of publication with the periodic distribution by 10-year interval is given in Fig. 3 .

### Area of Focus

**Fig. 4:**
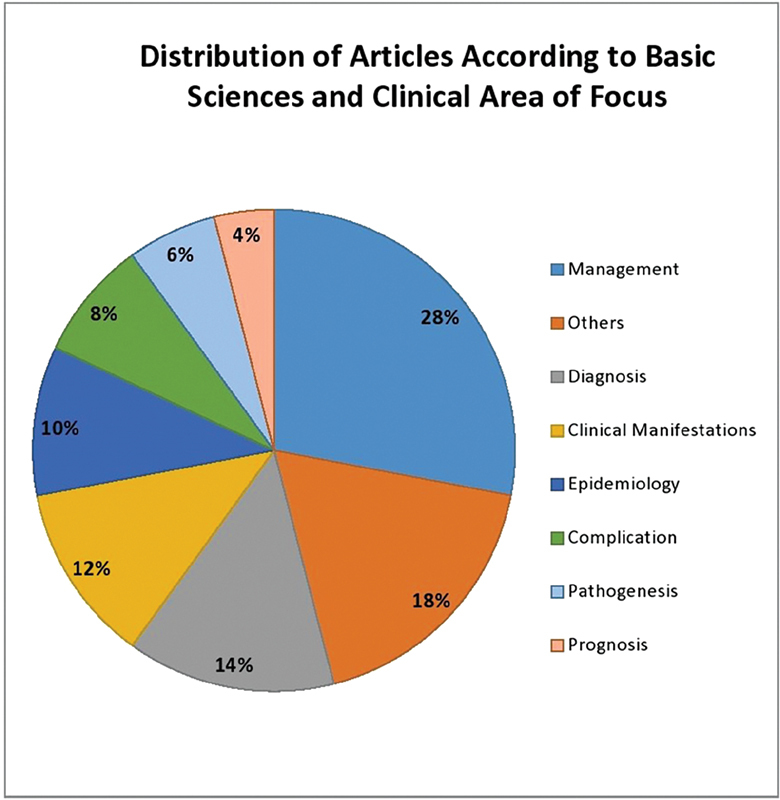
Distribution of articles on the basis of area of focus.

We've divided each extracted article on the basis of clinical or basic sciences aspect that have been the core objective of the GCA-related study. As show in Fig. 4 , majority of most influential articles on GCA were focused on Management of this illness (28%), followed by other articles (18%) and studies on diagnostic modalities (14%) of GCA. Prognosis-based studies (4%) constitute the least focused topic.

### Top Ranked Sources

Total 18 different journals contributed in the publication of most impactful articles on GCA out of which top-5 journals with 3 or more publications in presented in Table 1 . Majority of articles among top-50 most cited list (70%) belongs to these 5 journals which was topped by *Annals of Internal Medicine* with 13 publications. Among all the 18 journals, *New English Journal of Medicine (NEJM)* was the highest impact factor journal (impact factor of 70.60) but only had 2 publications from the top-50 list. However, *Annals of Internal Medicine* ( *n* = 13) and *Arthritis and Rheumatism* ( *n* = 12) had significantly more number of publications as compared with NEJM despite of a significant gap in impact factors. Pearson product-moment correlation coefficient test was used to assess relation between number of publication in top-50 list and journal's impact factor. There was no significant correlation (p = > 0.05) found between these two variables.

### Top Ranked Scholars

The highly ranked author based on the number of publications was *Hunder GG* ( *n* = 20) with h-index of 40, retaining affiliation with Mayo Clinic, Rochester, United States. However, despite having 20 articles out of top-50 list to his name, he was a principle author in only one of these articles. Because of *Hunder GG, Mayo Clinic* was the most frequently mentioned institute among the affiliations.

### Top Ranked Regions

Total of 11 different countries scholarly work contributed in the generation of most impactful articles list on GCA. The analysis proclaims United States to be the most productive country rendering most of the articles (64%) followed by Spain (16%) and United Kingdom (10%). We can compare and correlate our findings with other bibliometric analysis as United States is currently leading the marathon of quality research productivity. 12 Much notable point in Fig. 1 is the absence of any Asian or African country.

### Growth or Decline in Citation Frequency

Variability in citation frequency is an indicator of growth or decline in scholarly value of an article. 13 It is essential to continuously keep an eye on changing trends in citation collection. Fig. 2 in our study present the dataset of altering trends of top-50 most influential articles. It was observed that there was a significant rise in citation frequency of GCA-related articles during 1990s and 2000s decade. It has risen from 56 citations received by GCA most impactful article in year 1990 to 762 citations in year 2009.

### Impact of Self-citation

Self-citation is a bias which need to address in detail. Therefore, using Scopus tool for exclusion of self-citation, we extracted the dataset comparing total number of citation and citation count after excluding self-citations. The graphical picture of that dataset is presented in Fig. 2 . Paired *t* -test was applied to determine the mean difference between the frequencies of total citation obtained over the past 50 years versus frequencies of citation count extracted after excluding self-citation. There was a strong significant difference (p = < 0.001) found between the mean frequencies of two variables.

## Discussion

Giant cell arteritis has a slowly progressive debilitating course of illness which is difficult to predict. It usually presents with vague clinical and laboratory findings including headache, jaw claudication, scalp tenderness, raised C-reactive protein (CRP) and elevated erythrocyte sedimentation rate (ESR). However, the most distressing early ischemic complication of temporal arteritis is visual impairment (usually unilateral) which is also a presenting complain or immediately develop after diagnosis in ∼30% of the patients. Late and potentially life-threatening complications include thoracic/abdominal aortic aneurysms. 14 Early diagnosis and appropriate clinical management can prevent such dreadful complications. As represented in our Fig. 4 , studies included in the list of most influential top-50 articles on GCA were mainly focused on management of GCA (28%) followed by diagnostic strategies (14%) and clinical manifestations (12%).

According to a study conducted by Borchers AT et al and associates, incidence of GCA was at its peak (28.5) between the period of 1980 and 1985 in Olmsted County (USA). This incidence dropped to 21.4 in 1990–1994 and to 18.5 in 1995–2000. Similarly in Spain, the most significant decline in the incidence of GCA was observed between the decade of 1996–2005 (dropped from 15.9 to 12.92). 14 In our study, majority of articles (70%) in the top-50 list were published between the time period of 1990 and 2009 as represented in Fig. 3 . Other than that, United States (32 articles) and Spain (8 articles) were the major contributors in the most influential work on GCA ( Fig. 1 ). Back in time, the incidence of GCA was much significant in these two countries as compared with other regions of the globe. Considering the findings in our study and comparing it to the above mentioned study by Borchers AT, we can interpret that improved production and generation of high quality literature might have helped in controlling the incidence of GCA during that era, especially in USA and Spain.

In Fig. 3 , we can also observe that there is a substantial drop in influential scholarly production on GCA during last decade. It can be due to the fact that some novel but highly impactful researches couldn't make it to the top-50 list because of insufficient citation count. In a bibliometric analysis on asthma, it was mentioned that it takes a couple of years for an article after publication to start receiving citations on it. The citation frequency peak in a decade or two and begin to decline after that. 15 Our study also support this observation that a study usually receive most of its citation after 10–15 years of publication. Graphical representation of citation frequency through each decade show that the graphs were on continual rise during 1990s and 2000s decade ( Fig. 2 ). In our dataset, this observation can be infer as a prime reason behind the paucity of articles published between 2010 and 2019 to make it to the top-50 list on the basis of relatively low number of citations received so far.

If we discuss about the incidence and prevalence of temporal arteritis in Asian region, the only Asian country with an organized data on it is Japan, with a reported incidence of 1.47/100,000 people who are being treated for GCA. According to a retrospective review conducted at University of California-San Francisco (UCSF), a roughly estimated incidence of GCA in Asia population is between 0.09 and 1.5 per 100,000, which indicate a relatively lower risk of developing this disease as compared with Northern Europe and Northern United States, where the incidence of GCA diagnosed on the basis of temporal artery biopsy is between 6.9 and 29.1 per 100,000. 16 In this research, we can observe from Fig. 1 that there is no Asian country among the countries that contributed in most influential data on GCA. Almost all the articles in top-50 list has originated from either American or European region. This uneven distribution can be due to the fact that the incidence of GCA is territory-based with ethnicity playing a part in its incidents. Another explanation for this uneven distribution is a lack of proper diagnosis and organized database in certain geographic regions. One of the most recently published epidemiologic studies did not show a difference in the incidence of GCA based on race. 17 This contradicts previous studies, which reported higher incidences in white and particularly Scandinavian-origin ethnicities. 18

Temporal arteritis is relatively a ‘confined’ topic as compared with hypertension and diabetes. It doesn't usually co-exist with numerous other medical illnesses, not an epidemic disease and doesn't significantly influence the course of chronic metabolic disorders. Therefore, the citation count of most influential articles on giant cell arteritis were ranging from 187 to 598 times in our study. This number is substantially lower than the broad spectrum medical illnesses such as hypertension (582–7248) and diabetes (1121–10292). 19 20 This shows that citations differed between different medical specialties, mainly depending on the number of researchers who worked in specific medical fields and the specificity of each topic.

Bbibliometric analysis is widely accepted method to extract the list of most influential articles and authors within a field. However, few related bias raises a question on its credibility and accuracy. Those bias includes impact of self-citation, citation count of guidelines and ranking articles solely on the basis of highest number of citations. In this research, we've taken these factors into account and tried to remove it scientifically to minimize the risk of bias. Self-citation or self-referencing is an inappropriate and unjust method of improving the visibility and significance of an article or an author. Scientific literature provided some evidence that it doesn't potentially alter the total number of citations by much. A study on impact of self-citation on academic radiology concluded that it has almost negligible effect on H-index. 21 However, our findings contradict from this observation. In our study, we've found a significant difference between the means of total number of citation frequencies versus frequencies of citation count after excluding self-citations. Self-citation does inflict a considerable bias when analyzing citation classics and it is suggested to take it into account in future bibliometrics. Scopus provides a specialized programmed tool for such intervention and can be utilized to remarkably improve the accuracy of citation classics.

Naturally, clinical guidelines, classifications and grant supported studies are more frequently cited as compared with other form of articles because of an immense readership. 12 Additionally, due to the fact that most author discuss it for the purpose of ‘background check’, to illustrate the extent of work that has already been done on a specific topic. To overcome it, we've excluded clinical guidelines, classifications and grant supported researches from our study. Lastly, ranking of article solely on the basis of highest citation count generated some serious debate among scholarly community. To remove this bias in bibliometric analysis data, authors use ‘corrected ranks’ which is constructed by calculating average number of citations per year an article has received. 22 We've also calculated the corrected rank in our study which is given in Supplementary Table S1 (online only). With inclusion of this, we believe that the recent articles can also be valued according to their importance.

Tendency for top-cited articles to be published in general or specialized journals varies across different medical fields. According to Bradford's law, a small number core journal in the specialized field were mainly used to obtain citations; there were significantly lesser citations of articles published in non-core journals. 23 Thus, most of the highly cited articles publish in few major, specialized medical journals. We found in our study that majority of the top highly cited articles were published in specialized journals such as *Annals of Rheumatic diseases* and *Arthritis & Rheumatism* (continued as Arthritis and Rheumatology). This finding is in accordance with the suggested Bradford's law.

### Limitations

In the current study, we've conducted in-depth analysis on various metrics of top-50 most influential articles related to Giant cell arteritis (GCA). Nevertheless, this study presents some limitations. First, usage of only single bibliographic database in our research can result in a default pretermission of potentially impactful articles that are not indexed in Scopus. It is worth mentioning that Scopus does not index all the journals. Therefore, some publications may have been ignored and their number of citations may be underestimated. 24 But since Scopus covers more than 36,000 journals with majority of them having co-existing indexing in Medline and Embase as well, there is a lesser likelihood to miss out on significantly impactful researches. 25 Second, we've only considered articles written in English language because of which, we can miss out valuable data published in different native languages. Lastly, Garfield et al describe an ‘obliteration of incorporation’ effect in 1987. It describes the potential bias in a bibliometric study created by gradual loss of spotlight position of an older classic article which negatively affect their citation frequency. 5 However, this bias is trivial in our study because we've calculated the total number of citations for an article which includes both classic and novel citations.

## Conclusion

Our article identifies the strengths and weaknesses in medical literature contributions on Giant cell arthritis. It also identifies the prominent contributors and prominent publications on GCA. Most numbers of articles on GCA were published during the period of 2000–2009. The top source was found to be “ *Annals of Internal medicine* .” *Hunder GG* was found to be the most cited scholar and due to his contribution, his institute, Mayo clinic in Rochester, USA was the institute with most contributions. The United States was found to be the most active country in Giant cell arteritis research contributions. This bibliometric analysis on Giant cell arteritis publications has identified the information which may be utilized to direct future research contributions, identify field experts, guide researchers to fill knowledge gaps and assist in research fund allocation.

## References

[1] Weyand C M Goronzy J J Giant-cell arteritis and polymyalgia rheumatica Ann Intern Med 2003 139 06 505 515 13679329 10.7326/0003-4819-139-6-200309160-00015

[2] Salvarani C Cantini F Hunder G G Polymyalgia rheumatica and giant-cell arteritis Lancet 2008 372(9634):234 245 18640460 10.1016/S0140-6736(08)61077-6

[3] De Smit E Palmer A J Hewitt A W Projected worldwide disease burden from giant cell arteritis by 2050 J Rheumatol 2015 42 01 119 125 25362658 10.3899/jrheum.140318

[4] Agarwal A Durairajanayagam D Tatagari S Bibliometrics: tracking research impact by selecting the appropriate metrics Asian J Androl 2016 18 02 296 309 26806079 10.4103/1008-682X.171582 PMC4770502

[5] Garfield E 100 citation classics from the Journal of the American Medical Association JAMA 1987 257 01 52 59 3537352

[6] Gutiérrez-Salcedo M Some bibliometric procedures for analyzing and evaluating research fields Appl Intell 2018 48 05 1275 1287

[7] Tam W W Wong E L Wong F C Hui D S Citation classics: Top 50 cited articles in ‘respiratory system’ Respirology 2013 18 01 71 81 22978302 10.1111/j.1440-1843.2012.02262.x

[8] Ponce F A Lozano A M Highly cited works in neurosurgery. Part II: the citation classics J Neurosurg 2010 112 02 233 246 20078193 10.3171/2009.12.JNS091600

[9] O'Connor D J Gargiulo N J III Scher L A Jang J Lipsitz E C One hundred vascular surgery citation “classics” from the surgical literature J Vasc Surg 2011 53 04 1150 1156 20801612 10.1016/j.jvs.2010.06.158

[10] Elgafy H K Miller J D Hashmi S Ericksen S Top 20 cited Spine Journal articles, 1990-2009 World J Orthop 2014 5 03 392 397 25035845 10.5312/wjo.v5.i3.392 PMC4095035

[11] Murray M R Wang T Schroeder G D Hsu W K The 100 most cited spine articles Eur Spine J 2012 21 10 2059 2069 22526702 10.1007/s00586-012-2303-2 PMC3463701

[12] Shuaib W Khan M S Shahid H Valdes E A Alweis R Bibliometric analysis of the top 100 cited cardiovascular articles Am J Cardiol 2015 115 07 972 981 25670637 10.1016/j.amjcard.2015.01.029

[13] Nieminen P Carpenter J Rucker G Schumacher M The relationship between quality of research and citation frequency BMC Med Res Methodol 2006 6 01 42 16948835 10.1186/1471-2288-6-42 PMC1570136

[14] Borchers A T Gershwin M E Giant cell arteritis: a review of classification, pathophysiology, geoepidemiology and treatment Autoimmun Rev 2012 11(6-7):A544 A554 22285588 10.1016/j.autrev.2012.01.003

[15] Qu Y Zhang C Hu Z The 100 most influential publications in asthma from 1960 to 2017: A bibliometric analysis Respir Med 2018 137 206 212 29605206 10.1016/j.rmed.2018.03.014

[16] Pereira L S Yoon M K Hwang T N Giant cell arteritis in Asians: a comparative study Br J Ophthalmol 2011 95 02 214 216 20584707 10.1136/bjo.2009.177220

[17] Gruener A M Poostchi A Carey A R Association of giant cell arteritis with race JAMA Ophthalmol 2019 137 10 1175 1179 31393529 10.1001/jamaophthalmol.2019.2919 PMC6692689

[18] Gonzalez-Gay M A Vazquez-Rodriguez T R Lopez-Diaz M J Epidemiology of giant cell arteritis and polymyalgia rheumatica Arthritis Rheum 2009 61 10 1454 1461 19790127 10.1002/art.24459

[19] Oh Y S Galis Z S Anatomy of success: the top 100 cited scientific reports focused on hypertension research Hypertension 2014 63 04 641 647 24379185 10.1161/HYPERTENSIONAHA.113.02677 PMC3954897

[20] Zhao X Guo L Lin Y The top 100 most cited scientific reports focused on diabetes research Acta Diabetol 2016 53 01 13 26 26596851 10.1007/s00592-015-0813-1

[21] Rad A E Shahgholi L Kallmes D Impact of self-citation on the H index in the field of academic radiology Acad Radiol 2012 19 04 455 457 22285543 10.1016/j.acra.2011.11.013

[22] Fornaris-Cedeño Y Corrales-Reyes I E de Jesús Pérez-Martínez C Trigeminal neuralgia: bibliometric analysis of the fifty top-cited articles in the period 2000–2016 Journal of Oral Research 2018 7 07 245 254

[23] Brookes B C Bradford's law and the bibliography of science Nature 1969 224(5223):953 956 4902657 10.1038/224953a0

[24] Kulkarni A V Aziz B Shams I Busse J W Comparisons of citations in Web of Science, Scopus, and Google Scholar for articles published in general medical journals JAMA 2009 302 10 1092 1096 19738094 10.1001/jama.2009.1307

[25] Ramos M B Koterba E Rosi Júnior J Teixeira M J Figueiredo E G A Bibliometric Analysis of the Most Cited Articles in Neurocritical Care Research Neurocrit Care 2019 31 02 365 372 31087256 10.1007/s12028-019-00731-6

