# Supplementary material for: Bibliometric Analysis of Publication Activity in the Field of GIANT Cell Arteritis: A SCOPUS-based Study
Source: TH Open. 2022 Mar 10;6(1):e80–8. doi: 10.1055/a-1760-0340 (PMC8913176; doi:10.1055/a-1760-0340)
Supplement: Supplementary file 1 — Supplementary Material [file 10-1055-a-1760-0340-s220001.pdf]

Supplementary Table S1 List of top-50 most cited articles on Giant Cell Arteritis and related research

Rank	Article Detail	Number of Citations	Average Number of Citations per Year	Corrected Rank
1	Salvarani C, Cantini F, Boiardi L, Hunder GG. Polymyalgia rheumatica and giant-cell arteritis. N Engl J Med 2002;347(4):261–271	598	37.37	2
2	Schmidt WA, Kraft HE, Vorpahl K, Völker L, Gromnica-Ihle EJ. Color duplex ultrasonography in the diagnosis of temporal arteritis. N Engl J Med 1997;337(19):1336–1342	495	23.57	7
3	Chuang TY, Hunder GG, Ilstrup DM, Kurland LT. Polymyalgia rheumatica: a 10-year epidemiologic and clinical study. Ann Intern Med 1982;97 (5):672–680	479	13.3	26
4	Huston KA, Hunder GG, Lie JT, Kennedy RH, Elveback LR. Temporal arteritis: a 25-year epidemiologic, clinical, and pathologic study. Ann Intern Med 1978;88(2):162–167	472	11.8	30
5	Salvarani C, Cantini F, Hunder GG. Polymyalgia rheumatica and giant-cell arteritis. Lancet 2008;372(9634):234–245	445	44.5	1
6	Evans JM, O'Fallon WM, Hunder GG. Increased incidence of aortic aneurysm and dissection in giant cell (temporal) arteritis. A population-based study. Ann Intern Med 1995;122(7):502–507	436	18.95	15
7	Klein RG, Hunder GG, Stanson AW, Sheps SG. Large artery involvement in giant cell (temporal) arteritis. Ann Intern Med 1975;83(6):806–812	398	9.25	38
8	Hoffman GS, Cid MC, Rendt-Zagar KE, et al; Infliximab-GCA Study Group. Infliximab for maintenance of glucocorticosteroid-induced remission of giant cell arteritis: a randomized trial. Ann Intern Med 2007;146(9):621–630	360	32.72	3
9	Hoffman GS, Cid MC, Hellmann DB, et al; International Network for the Study of Systemic Vasculitides. A multicenter, randomized, double-blind, placebo-controlled trial of adjuvant methotrexate treatment for giant cell arteritis. Arthritis Rheum 2002;46(5):1309–1318	358	22.37	9
10	Hayreh SS, Podhajsky PA, Zimmerman B. Ocular manifestations of giant cell arteritis. Am J Ophthalmol 1998;125(4):509–520	351	17.55	20
11	Jover JA, Hernández-García C, Morado IC, Vargas E, Bañares A, Fernández-Gutiérrez B. Combined treatment of giant-cell arteritis with methotrexate and prednisone. a randomized, double-blind, placebo-controlled trial. Ann Intern Med 2001;134(2):106–114	344	20.23	14
12	Nuenninghoff DM, Hunder GG, Christianson TJH, McClelland RL, Matteson EL. Incidence and predictors of large-artery complication (aortic aneurysm, aortic dissection, and/or large-artery stenosis) in patients with giant cell arteritis: a population-based study over 50 years. Arthritis Rheum 2003;48(12):3522–3531	338	22.53	8
13	Brack A, Martinez-Taboada V, Stanson A, Goronzy JJ, Weyand CM. Disease pattern in cranial and large-vessel giant cell arteritis. Arthritis Rheum 1999;42(2):311–317	336	17.68	19
14	Weyand CM, Hicok KC, Hunder GG, Goronzy JJ. Tissue cytokine patterns in patients with polymyalgia rheumatica and giant cell arteritis. Ann Intern Med 1994;121(7):484–491	321	13.37	25
15	Proven A, Gabriel SE, Orces C, O'Fallon WM, Hunder GG. Glucocorticoid therapy in giant cell arteritis: duration and adverse outcomes. Arthritis Rheum 2003;49(5):703–708	320	21.33	10
16	Weyand CM, Goronzy JJ. Giant-cell arteritis and polymyalgia rheumatica. Ann Intern Med 2003;139(6):505–515	317	21.13	11
17	Mahr AD, Jover JA, Spiera RF, et al. Adjunctive methotrexate for treatment of giant cell arteritis: an individual patient data meta-analysis. Arthritis Rheum 2007;56(8):2789–2797	304	27.63	5

 © 2022. The Author(s).

Supplementary Table S1 (*Continued*)

Rank	Article Detail	Number of Citations	Average Number of Citations per Year	Corrected Rank
18	Wilkinson IMS, Russell RWR. Arteries of the head and neck in giant cell arteritis. A pathological study to show the pattern of arterial involvement. Arch Neurol 1972;27(5):378–391	304	6.61	45
19	Nesher G, Berkun Y, Mates M, Baras M, Rubinow A, Sonnenblick M. Low-dose aspirin and prevention of cranial ischemic complications in giant cell arteritis. Arthritis Rheum 2004;50(4):1332–1337	284	20.28	12
20	Smetana GW, Shmerling RH. Does this patient have temporal arteritis? JAMA 2002;287(1):92–101	284	17.75	18
21	Blockmans D, Stroobants S, Maes A, Mortelmans L. Positron emission tomography in giant cell arteritis and polymyalgia rheumatica: evidence for inflammation of the aortic arch. Am J Med 2000;108(3):246–249	283	15.72	22
22	Achkar AA, Lie JT, Hunder GG, O'Fallon WM, Gabriel SE. How does previous corticosteroid treatment affect the biopsy findings in giant cell (temporal) arteritis? Ann Intern Med 1994;120(12):987–992	283	11.79	31
23	González-Gay MA, García-Porrúa C, Llorca J, et al. Visual manifestations of giant cell arteritis. Trends and clinical spectrum in 161 patients. Medicine (Baltimore) 2000;79(5):283–292	281	15.61	23
24	Caselli RJ, Hunder GG, Whisnant JP. Neurologic disease in biopsy-proven giant cell (temporal) arteritis. Neurology 1988;38(3):352–359	281	9.36	37
25	Hayreh SS, Podhajsky PA, Raman R, Zimmerman B. Giant cell arteritis: validity and reliability of various diagnostic criteria. Am J Ophthalmol 1997;123(3):285–296	275	13	27
26	Salvarani C, Gabriel SE, O'Fallon WM, Hunder GG. The incidence of giant cell arteritis in Olmsted County, Minnesota: apparent fluctuations in a cyclic pattern. Ann Intern Med 1995;123(3):192–194	273	11.86	29
27	González-Gay MA, Blanco R, Rodríguez-Valverde V, et al. Permanent visual loss and cerebrovascular accidents in giant cell arteritis: predictors and response to treatment. Arthritis Rheum 1998;41(8):1497–1504	271	13.55	24
28	Aiello PD, Trautmann JC, McPhee TJ, Kunselman AR, Hunder GG. Visual prognosis in giant cell arteritis. Ophthalmology 1993;100(4):550–555	259	10.36	33
29	Healey LA. Long-term follow-up of polymyalgia rheumatica: evidence for synovitis. Semin Arthritis Rheum 1984;13(4):322–328	259	7.62	43
30	Roche NE, Fulbright JW, Wagner AD, Hunder GG, Goronzy JJ, Weyand CM. Correlation of interleukin-6 production and disease activity in polymyalgia rheumatica and giant cell arteritis. Arthritis Rheum 1993;36(9):1286–1294	253	10.12	24
31	Machado EBV, Michet CJ, Ballard DJ, et al. Trends in incidence and clinical presentation of temporal arteritis in Olmsted County, Minnesota, 1950-1985. Arthritis Rheum 1988;31(6):745–749	251	8.36	40
32	Gonzalez-Gay MA, Vazquez-Rodriguez TR, Lopez-Diaz MJ, et al. Epidemiology of giant cell arteritis and polymyalgia rheumatica. Arthritis Rheum 2009;61(10):1454–1461	249	26.66	6
33	Blockmans D, de Ceuninck L, Vanderschueren S, Knockaert D, Mortelmans L, Bobbaers H. Repetitive 18F-fluorodeoxyglucose positron emission tomography in giant cell arteritis: a prospective study of 35 patients. Arthritis Rheum 2006;55(1):131–137	243	20.25	13
34	Karassa FB, Matsagas MI, Schmidt WA, Ioannidis JPA. Meta-analysis: test performance of ultrasonography for giant-cell arteritis. Ann Intern Med 2005;142(5):359–369	237	18.23	17
35	De Silva M, Hazleman BL. Azathioprine in giant cell arteritis/polymyalgia rheumatica: a double-blind study. Ann Rheum Dis 1986;45(2):136–138	234	7.31	44

(*Continued*)

 © 2022. The Author(s).

Supplementary Table S1 (*Continued*)

Rank	Article Detail	Number of Citations	Average Number of Citations per Year	Corrected Rank
36	Hamilton CR Jr, Shelley WM, Tumulty PA. Giant cell arteritis: including temporal arteritis and polymyalgia rheumatica. Medicine (Baltimore) 1971;50(1):1–27	231	8.55	39
37	Klein RG, Campbell RJ, Hunder GG, Carney JA. Skip lesions in temporal arteritis. Mayo Clin Proc 1976;51(8):504–510	226	5.38	47
38	Fauchald P, Rygvold O, Oystese B. Temporal arteritis and polymyalgia rheumatica. Clinical and biopsy findings. Ann Intern Med 1972;77(6):845–852	222	4.82	49
39	Spiera RF, Mitnick HJ, Kupersmith M, et al. A prospective, double-blind, randomized, placebo controlled trial of methotrexate in the treatment of giant cell arteritis (GCA). Clin Exp Rheumatol 2001;19(5):495–501	209	12.29	28
40	Weyand CM, Fulbright JW, Hunder GG, Evans JM, Goronzy JJ. Treatment of giant cell arteritis: interleukin-6 as a biologic marker of disease activity. Arthritis Rheum 2000;43(5):1041–1048	207	11.5	32
41	Hunder GG, Sheps SG, Allen GL, Joyce JW. Daily and alternate-day corticosteroid regimens in treatment of giant cell arteritis: comparison in a prospective study. Ann Intern Med 1975;82(5):613–618	207	4.81	50
42	Jones JG, Hazleman BL. Prognosis and management of polymyalgia rheumatica. Ann Rheum Dis 1981;40(1):1–5	205	5.54	46
43	Evans JM, Bowles CA, Bjornsson J, Mullany CJ, Hunder GG. Thoracic aortic aneurysm and rupture in giant cell arteritis. A descriptive study of 41 cases. Arthritis Rheum 1994;37(10):1539–1547	194	8.08	41
44	Bengtsson BÅ, Malmvall BE. The epidemiology of giant cell arteritis including temporal arteritis and polymyalgia rheumatica. Arthritis &amp. Rheumatism 1981;24(7):899–904	194	5.24	48
45	Mazlumzadeh M, Hunder GG, Easley KA, et al. Treatment of giant cell arteritis using induction therapy with high-dose glucocorticoids: a double-blind, placebo-controlled, randomized prospective clinical trial. Arthritis Rheum 2006;54(10):3310–3318	192	16	21
46	Hayreh SS, Podhajsky PA, Zimmerman B. Occult giant cell arteritis: ocular manifestations. Am J Ophthalmol 1998;125(4):521–526	190	9.5	36
47	Liu GT, Glaser JS, Schatz NJ, Smith JL. Visual morbidity in giant cell arteritis. Clinical characteristics and prognosis for vision. Ophthalmology 1994;101 (11):1779–1785	189	7.87	42
48	Martínez-Taboada VM, Rodríguez-Valverde V, Carreño L, et al. A double-blind placebo controlled trial of etanercept in patients with giant cell arteritis and corticosteroid side effects. Ann Rheum Dis 2008;67(5):625–630	188	18.8	16
49	Unizony S, Arias-Urdaneta L, Miloslavsky E, et al. Tocilizumab for the treatment of large-vessel vasculitis (giant cell arteritis, Takayasu arteritis) and polymyalgia rheumatica. Arthritis Care Res (Hoboken) 2012;64 (11):1720–1729	187	31.16	4
50	Blockmans D, Maes A, Stroobants S, et al. New arguments for a vasculitic nature of polymyalgia rheumatica using positron emission tomography. Rheumatology (Oxford) 1999;38(5):444–447	187	9.84	35

 © 2022. The Author(s).
